# A ratiometric fluorescent probe for detection of exogenous mitochondrial SO_2_ based on a FRET mechanism†

**DOI:** 10.1039/c8ra10328c

**Published:** 2019-03-18

**Authors:** Zhiyang Xu, Zhen Chen, Aikun Liu, Ruixue Ji, Xiaoqun Cao, Yanqing Ge

**Affiliations:** School of Chemistry and Pharmaceutical Engineering, Taishan Medical University Tai'an 271000 PR China geyanqing2016@126.com +86-538-6229741

## Abstract

A novel imidazo[1,5-*a*]pyridine-hemicyanine based ratiometric fluorescent probe for detection of mitochondrial SO_2_ was designed and synthesized. The probe is based on a fluorescence resonance energy transfer (FRET) mechanism. It exhibits high selectivity and sensitivity towards SO_3_^2−^ with a fast response time (3 min) and detection limit of 0.13 μM. Further, it showed low cytotoxicity and was successfully applied to image exogenous mitochondrial SO_2_ in cells.

## Abstract 2

A novel imidazo[1,5-*a*]pyridine-hemicyanine based ratiometric fluorescent probe for detection of mitochondrial SO_2_ was designed and synthesized.

## Graphical abstract

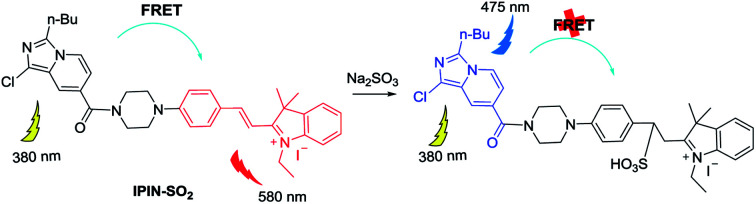

## Introduction

Sulfur dioxide (SO_2_), which used to be considered as a toxic environmental pollutant, is now considered to be a new possible signal molecule following nitric oxide, carbon monoxide and hydrogen sulfide.^1–4^ Cancers, neurological disorders and cardiovascular diseases could be caused by high exogenous SO_2_ levels. As exogenous SO_2_ is produced *via* oxidation of some sulphur-containing amino acids and hydrogen sulfide in mitochondria,^5,6^ it is extremely important to develop selective, sensitive and rapid methods for SO_2_ detection in mitochondria.

When hydrated in aqueous media, SO_2_ can be transformed into its derivatives bisulfite (HSO_3_^−^) and sulfite (SO_3_^2−^). Therefore, methods for the detection of HSO_3_^−^/SO_3_^2−^ such as electrochemistry, chromatography, titration and capillary electrophoresis have been developed.^7–10^ However, those methods can not realize imaging in cells.

Since the first sulfite fluorescent probe was reported by Chang group in 2010,^11^ numerous probes based on nucleophilic reactions with aldehydes, Michael additions, dequenching of levulinate and coordinative interactions have been developed in recent years.^12–29^ Despite the remarkable progress achieved, limitations such as long detection times and poor water solubility still remain. More importantly, those intensity-based probes are susceptible to factors like external environment, substrate concentration and instrument sensitivity.

Ratiometric fluorescent probes are more advantageous than intensity-based ones. Förster resonance energy transfer (FRET) mechanism is most widely used to construct well-performing ratiometric probes.^30–32^ To date, some well-behaved ratiometric fluorescent probes for SO_2_ derivatives have been developed.^33–36^ However, there is great room for improvement since these probes are still subject to some drawbacks such as unsatisfactory detection limits, long response time, and poor selectivity over H_2_S. Recently, we successfully synthesized the imidazole[1,5-*a*]pyridine *via* a tandem reaction.^37–39^ Some fluorescent probes based on this new fluorophore for Cu^2+^, and Hg^2+^ have been constructed subsequently.^40,41^ Continuing our efforts to search for new fluorophore and extend their applications,^42–53^ herein, we report a new FRET platform for the rapid detection of SO_2_. Imidazole[1,5-*a*]pyridine was selected as donor, hemicyanine dyad as receptor, and piperazine as connection unit. The probe IPIN-SO_2_ can detect SO_3_^2−^ rapidly (3 min) and sensitively in a wide pH range of 5–10. More importantly, IPIN-SO_2_ can be used for imaging exogenous mitochondrial SO_2_ in cells.

## Experimental

### Materials and apparatus

UV-vis spectra were recorded on a U-2600 UV-vis spectrometer (Hitachi) and fluorescence spectra were recorded on a RF-5301PC luminescence spectrophotometer (Shimadzu) at room temperature. ^1^H NMR and ^13^C NMR spectra were measured on a Bruker Avance 400 (400 MHz) spectrometer (CDCl_3_ as solvent and tetramethylsilane (TMS) as an internal standard). HRMS spectra were recorded on a Q-TOF6510 spectrograph (Agilent). Nikon fluorescence inverted microscope (Ti 2-U) was used to record cell imaging. All reagents and solvents were purchased from commercial sources and used without further purification. Metal ion solution was prepared by dissolving the deionized water with metal chloride as raw material. The anionic solution was prepared by dissolving sodium containing compounds into deionized water. Deionized water was used in the whole absorption and fluorescence detection process.

### Cell culture and imaging

Brain glioma cells were cultured in RPMI-1640 containing 10% bovine serum in a 5% CO_2_/95% air incubator at 37 °C. For cell imaging experiments, the growth medium was removed and replaced by RPMI-1640 without CS. The cells were incubated in a 1 μM IPIN-SO_2_ incubator at 37 °C and 5% CO_2_/95% air for 30 minutes. The cells were washed with PBS three times and cell images were obtained *via* an inverted fluorescence microscope from Ti 2-U (Nikon, ECLIPSE, equipped with FRET system, Mercury lamp light source). For the probe, the excitation light source is 395/25 nm and the emission collected through filter is 605/55 nm and 460/50 nm. The colocalization experiments have been carried out through Laser Scanning Confocal Microscope (FV1000, Olympus). For the probe, the excitation light source is 405 nm and the emission collected is 560–620 nm. For the MitoTracker@ Deep Red, the excitation light source is 633 nm and the emission collected through filter is 650–700 nm.

### Synthesis

#### Synthesis of compound 3

Compound 1 and 2 were synthesized according to the literature.^5^

Compound 1 (315 mg, 1 mmol) was dissolved in ethanol (15 mL), then compound 2 (190 mg, 1 mmol) was added, and 3 drops of piperidine were added. After heating and refluxing for 12 hours, the solvent was removed under reduced pressure. A deep red solid was obtained which was used for the next step without further purification.

#### Synthesis of the probe IPIN-SO_2_

Compound 4 were synthesized according to the literature.^33^

Compound 4 (253 mg, 1 mmol) was added to 30 mL dichloromethane and then DMAP (183 mg, 1.5 mmol) and EDC (288 mg, 1.5 mmol) were added. After stirring at room temperature for half an hour, compound 3 (488 mg, 1 mmol) was added and stirred for 12 hours at room temperature. Then the solvent was removed under reduced pressure to afford crude compound IPIN-SO_2_, which was purified on a silica gel column (C_2_H_5_OH : CH_2_Cl_2_ = 1 : 100; yields: 68.9%). ^1^H NMR (400 MHz, CDCl_3_) *δ* 8.20 (d, *J* = 8.0 Hz, 2H), 8.09 (d, *J* = 12.0 Hz, 1H), 7.76 (d, *J* = 8.0 Hz, 1H), 7.58–7.50 (m, 7H), 7.02 (d, *J* = 8.0 Hz, 2H), 6.72 (d, *J* = 8.0 Hz, 1H), 4.86 (q, *J* = 8.0 Hz, 2H), 3.86 (s, 4H), 3.66 (s, 4H), 2.97 (t, *J* = 8.0 Hz, 2H), 1.81 (m, 6H), 1.60 (m, 5H), 1.44 (m, 2H), 0.97 (t, *J* = 8.0 Hz, 3H). ^13^C NMR (100 MHz, CDCl_3_) *δ* 179.3, 167.6, 154.9, 154.6, 142.6, 140.6, 138.7, 135.2, 129.5, 128.5, 124.1, 122.6, 120.9, 117.9, 114.2, 113.4, 112.3, 106.8, 51.4, 46.7, 44.5, 42.9, 29.0, 27.6, 26.3, 22.5, 22.0, 14.0, 13.8. HRMS: ([M]^+^); calcd for C_36_H_41_ClN_5_O: 594.2994; found: 594.3003.

## Results and discussion

### Synthesis of the probe IPIN-SO_2_

**Scheme 1:**
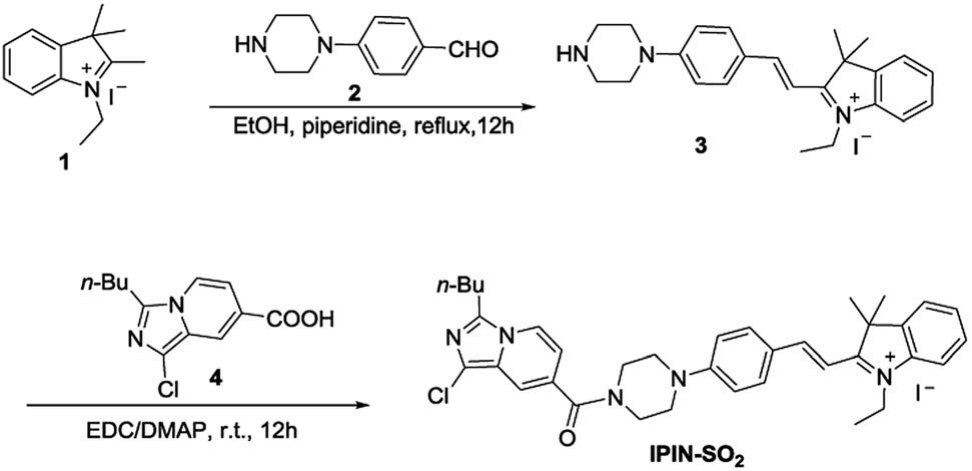
Synthesis of probe IPIN-SO_2_.

The synthetic route of probe IPIN-SO_2_ is shown in Scheme 1. Probe IPIN-SO_2_ was obtained as deep purple solid powder in 89.5% yield through classical condensation of compound 3 and compound 4. The probe IPIN-SO_2_ was characterized by ^1^H NMR, ^13^C NMR and HRMS.

### UV-vis and fluorescence spectra response of IPIN-SO_2_

**Fig. 1:**
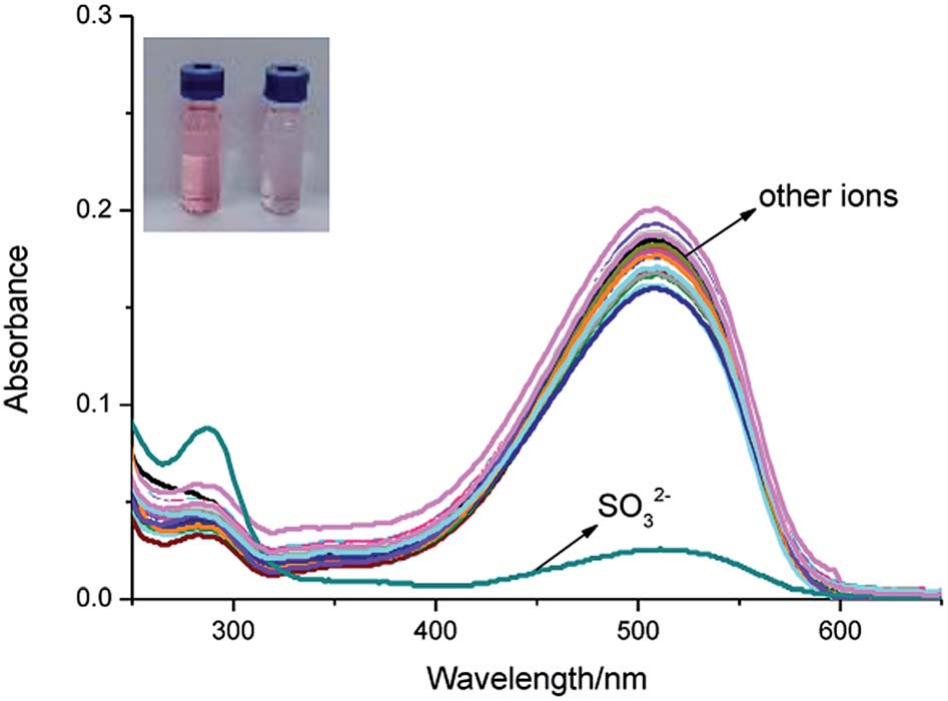
Ultraviolet absorption spectra of IPIN-SO_2_ (10 μM) in 0.1 M PBS (pH = 7.4) buffer solution with addition of 100 μM of various species (SO_3_^2−^, AcO^−^, Br^−^, H_2_PO_4_^−^, Cl^−^, CO_3_^2−^, HCO_3_^−^, F^−^, HPO_4_^2−^, I^−^, NO_2_^−^, NO_3_^−^, S_2_O_3_^2−^, SO_4_^2−^, HS^−^, Fe^3+^, Ca^2+^, Cu^2+^, K^+^, Na^+^, Zn^2+^), GSH (glutathione), Hcy (homocysteine), Cys (cysteine).

As shown in Fig. 1, IPIN-SO_2_ is more selective to SO_3_^2−^ compared with other competitive ions, which do not cause any significant absorption changes in the visible region, which can be used as a “naked eye” chemical colorimeter. When added 10 equiv. SO_3_^2−^, the solution was obviously lighter from pink (Fig. 1, inset).

**Fig. 2:**
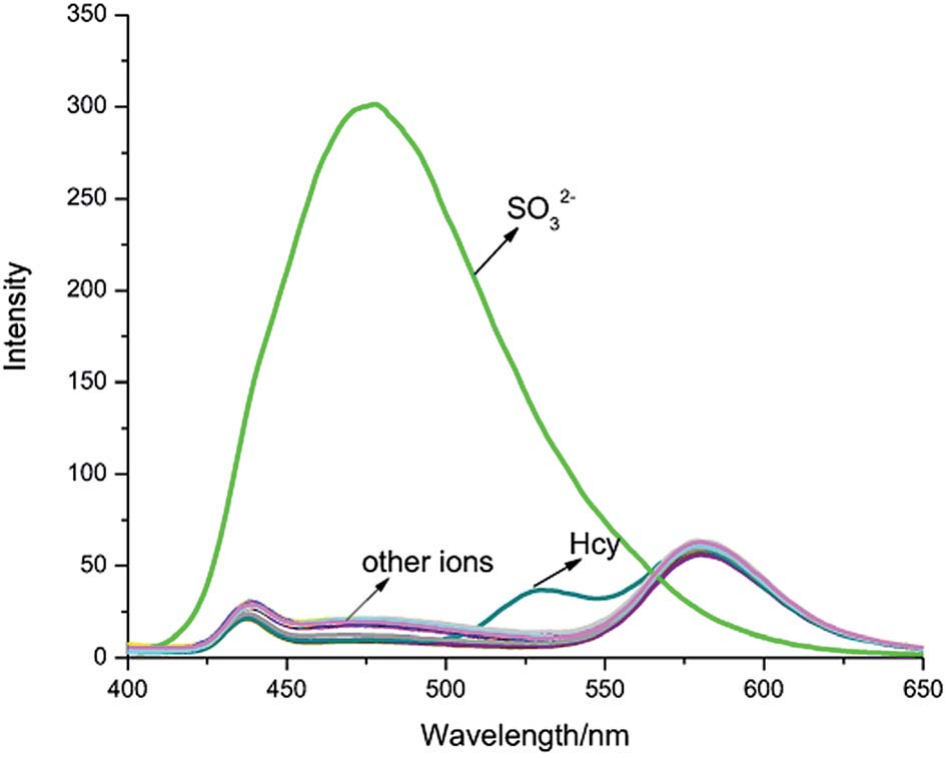
Fluorescence spectra of IPIN-SO_2_ (10 μM) in 0.1 M PBS (pH = 7.4) buffer solution with addition of 100 μM of various species (*λ*_ex_ = 380 nm).

The interaction between IPIN-SO_2_ and SO_3_^2−^ was further studied by UV-vis spectroscopic titration in 0.1 M PBS (pH = 7.4) buffer solution. IPIN-SO_2_ showed characteristic absorption at 500 nm. However, when the sulfite was added to the IPIN-SO_2_ solution, the absorption peak at 500 nm decreased rapidly with the increase of sulfite concentration as shown in Fig. S1.† When 10 equiv. amount of SO_3_^2−^ was added to IPIN-SO_2_ solution, the fluorescence intensity immediately increased at 475 nm and the fluorescence intensity at 580 nm decreased significantly. By contrast, the other competitive cations did not cause any significant fluorescence changes, indicating that IPIN-SO_2_ has a better selectivity for SO_3_^2−^ in the fluorescence spectrum as shown in Fig. 2.

**Fig. 3:**
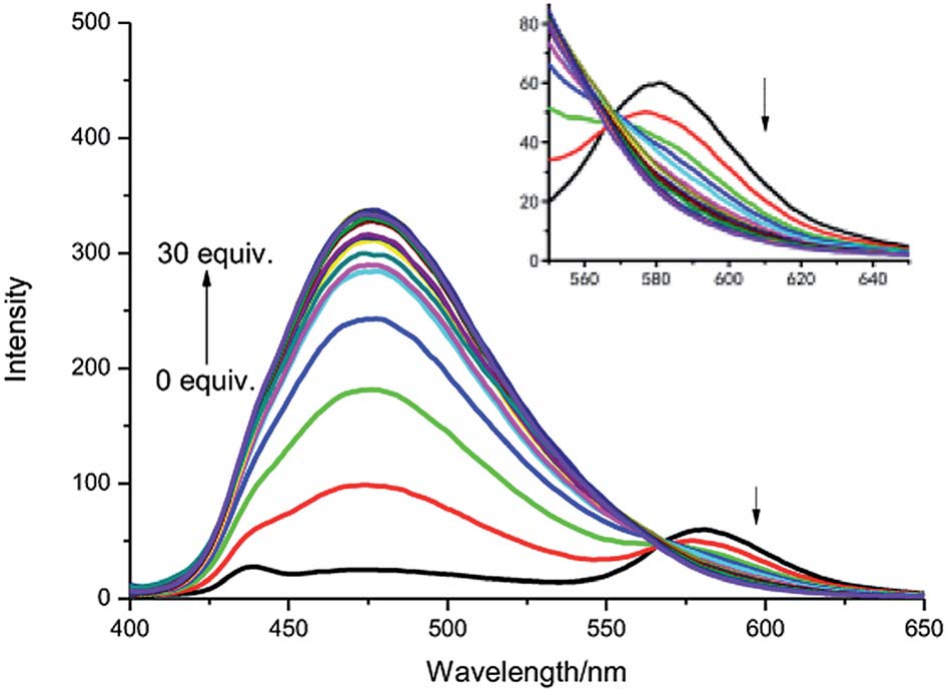
Fluorescence spectra of IPIN-SO_2_ (10 μM) with the addition of SO_3_^2−^ (0–30 equiv.) in 0.1 M PBS (pH = 7.4) buffer solution (*λ*_ex_ = 380 nm).

As shown in Fig. 3, the fluorescence titration process of IPIN-SO_2_ was recorded. In IPIN-SO_2_ aqueous solution, with the increase of SO_3_^2−^ concentration, the fluorescence intensity increases at 475 nm and decreases at 580 nm, and the two emission peaks can be well separated (105 nm). The results show that the developed FRET system can effectively avoid the overlap of emission spectra and ensure the high resolution and accuracy of the determination. Moreover, when SO_3_^2−^ concentration increased from 0 μM to 30 μM, the fluorescence intensity ratio increased from 0.29 to 12.78, about 44 times. When SO_3_^2−^ concentration was in the range of 1.5–4.0 μM, there was a good linear relationship as shown in Fig. S2.†

According to LOD = 3*σ*/*k* (*σ* is the standard deviation of ten blank solutions and *k* is the slope of the linear calibration plot between the fluorescence intensity and the concentration of SO_3_^2−^), the detection limit was as low as 0.13 μM.

**Fig. 4:**
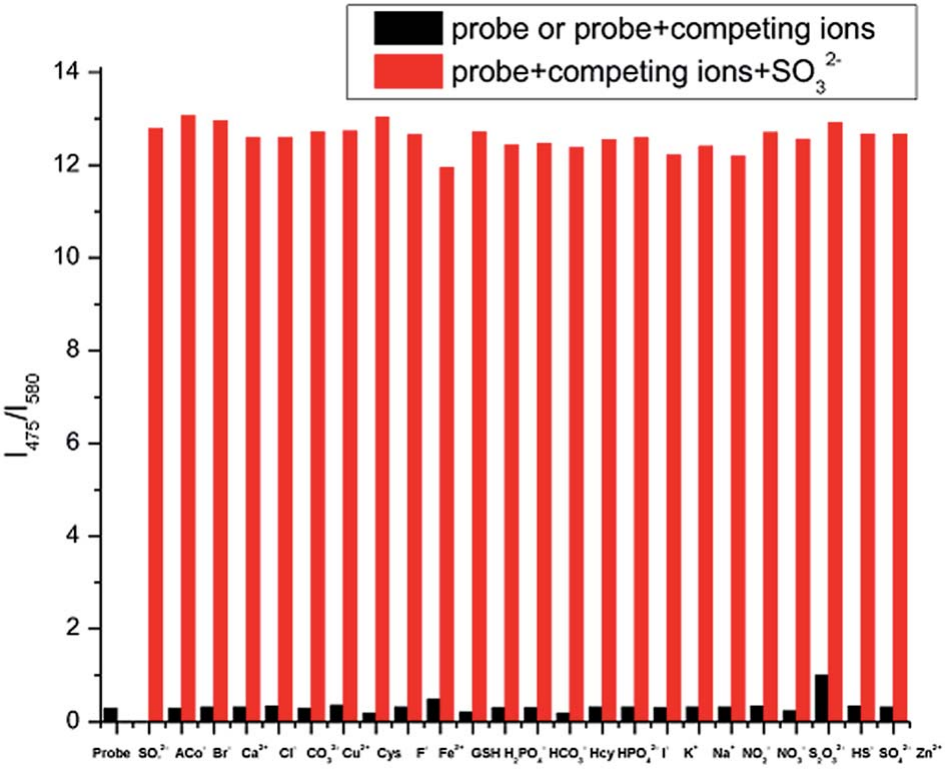
Ratiometric fluorescence responses *I*_475_/*I*_580_ of IPIN-SO_2_ (10 μM) upon the addition of 10 equiv. SO_3_^2−^ in the presence of 100 μM background ions in PBS (pH = 7.4) buffer solution.

In addition, the interference experiments were carried out under the coexistence of various species (Fig. 4). Background ions did not interfere with fluorescence intensity. Sulfite-induced fluorescence enhancement (*I*_475_/*I*_580_) remained unaffected by the coexistence of other species.

### Kinetic study

**Fig. 5:**
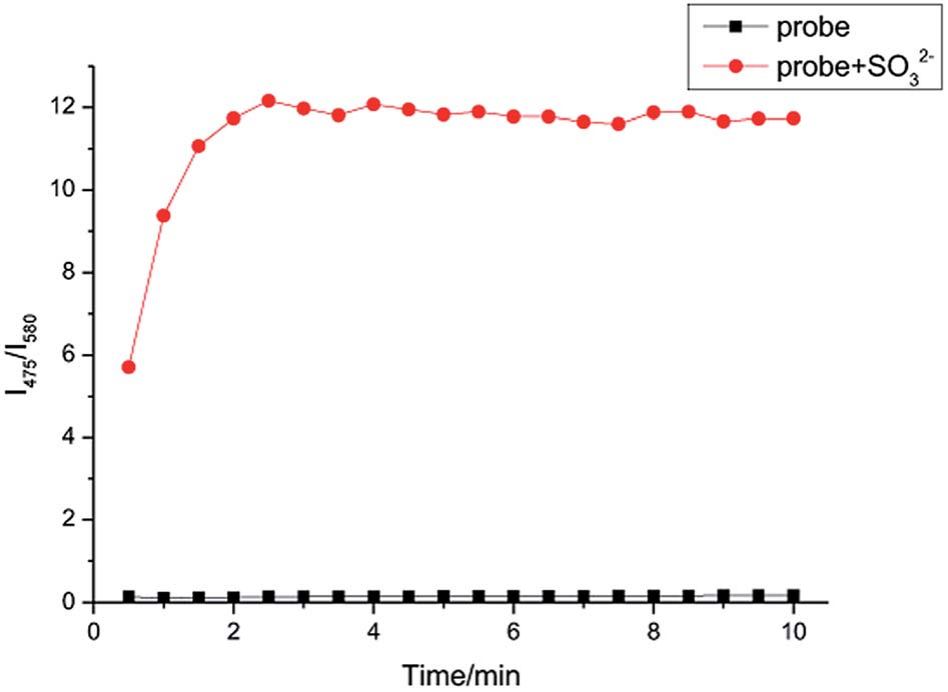
Time dependent increase of IPIN-SO_2_ (10 μM) fluorescence intensities after addition of 10 equiv. SO_3_^2−^ in PBS (pH = 7.4) solution (*λ*_ex_ = 380 nm).

In Fig. 5, the time course of fluorescence response of IPIN-SO_2_ aqueous solution with SO_3_^2−^ is shown. The fluorescence intensity ratio (*I*_475_/*I*_580_) reached the maximum value in 3 min when 10 equiv. SO_3_^2−^ was added and the fluorescence ratio of IPIN-SO_2_ almost remained unchanged with time, which indicates that IPIN-SO_2_ can be used as a fast response SO_3_^2−^ probe.

### Effect of pH

**Fig. 6:**
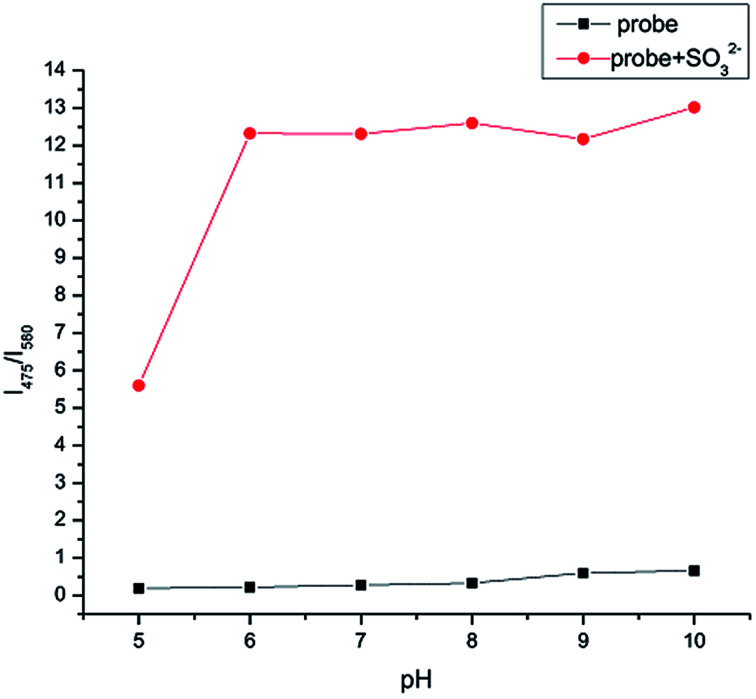
Ratio of fluorescent intensities at 475 nm and 580 nm for IPIN-SO_2_ (10 μM) in the presence of SO_3_^2−^ (10 equiv.) at varied pH values (*λ*_ex_ = 380 nm).

As shown in Fig. 6, in order to detect SO_3_^2−^ efficiently and selectively, the effect of different acid concentrations on IPIN-SO_2_ was studied to find the suitable pH range. In PBS buffer solution, the fluorescence titration curves of IPIN-SO_2_ and IPIN-SO_2_ have no obvious change between pH 5.0 and 10.0 of fluorescence intensity ratio (*I*_475_/*I*_580_), indicating that the sensor IPIN-SO_2_ and the sensor IPIN-SO_2_ existing in SO_3_^2−^ are stable within this pH range.

### Mechanism

**Scheme 2:**
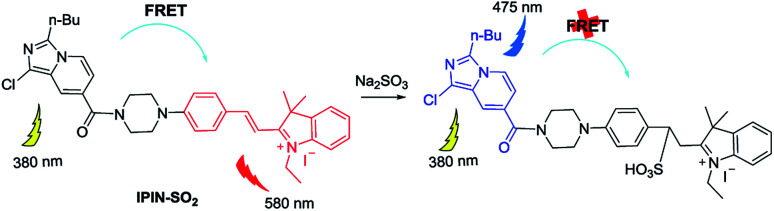
Proposed sensing mechanism of IPIN-SO_2_ with SO_3_^2−^.

As shown in Scheme 2, once the IPIN-SO_2_ energy donor is excited, FRET will enter the hemicyanine group from the imidazole[1,5-*a*]pyridine fluorescent group, which may weaken or even quench the fluorescence of the imidazole[1,5-*a*]pyridine fluorescent group. Interruption of the p–π conjugation in the hemicyanine fluorophore results in increasing the energy of its first singlet level above that of the donor group. In addition, both the probe IPIN-SO_2_ and the donor can be activated at 380 nm, but the receptor cannot, which further confirms the FRET process in probe IPIN-SO_2_.

To further clarify the proposed mechanism, HRMS of the reaction product was conducted. A clear mass (*m*/*z* 676.2735) of adduct appeared after the probe reacted with SO_3_^2−^ (Fig. S8†).

### Cell imaging

**Fig. 7:**
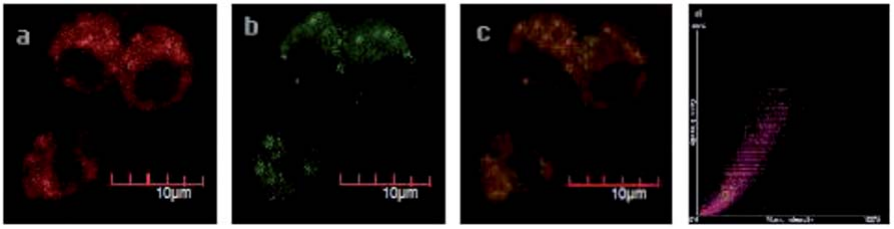
Glioma cells were incubated with IPIN-SO_2_ (0.1 μM) for 1 h, followed by MitoTracker@ Deep Red (0.01 μM) for 0.5 h. (a) The fluorescence image of MitoTracker@ Deep Red FM. *λ*_ex_ = 633 nm, *λ*_em_ = 650–700 nm. (b) The fluorescence image of probe IPIN-SO_2_. *λ*_ex_ = 405 nm, *λ*_em_ = 560–620 nm. The red fluorescence was colored as green for discrimination. (c) Merged images of (a) and (b). (d) Colocalization coefficient (Pearson's coefficient) of IPIN-SO_2_ and MitoTracker@ Deep Red was 0.948.

As probe IPIN-SO_2_ shows excellent optical response to SO_3_^2−^*in vitro*, cell imaging of IPIN-SO_2_ has been further studied in glioma cells. IPIN-SO_2_ fluorescence is stable in living cell (Fig. S3†) and cytotoxicity is negligible at 1–16 μM concentration (Fig. S4†). Because cationic cyanine dyes may accumulate in mitochondria,^54,55^ colocalization assays were performed with MitoTracker@ Deep Red FM and IPIN-SO_2_ (Fig. 7). The fluorescence of IPIN-SO_2_ and MitoTracker@ Deep Red FM has a significant overlap, and the overlap coefficient is 0.948 (Fig. 7d), indicating that IPIN-SO_2_ was well distributed in mitochondria.

Then the probe IPIN-SO_2_ was applied to SO_3_^2−^ imaging in living glioma cells. When the glioma cells were incubated with probe IPIN-SO_2_ for 1 hour, the red channel showed strong fluorescence and blue channel weak fluorescence (Fig. S5†). When incubated for 0.5 hour with different concentrations of Na_2_SO_3_, the cells showed enhanced fluorescence of red channel and decreased fluorescence of blue channel.

**Fig. 8:**
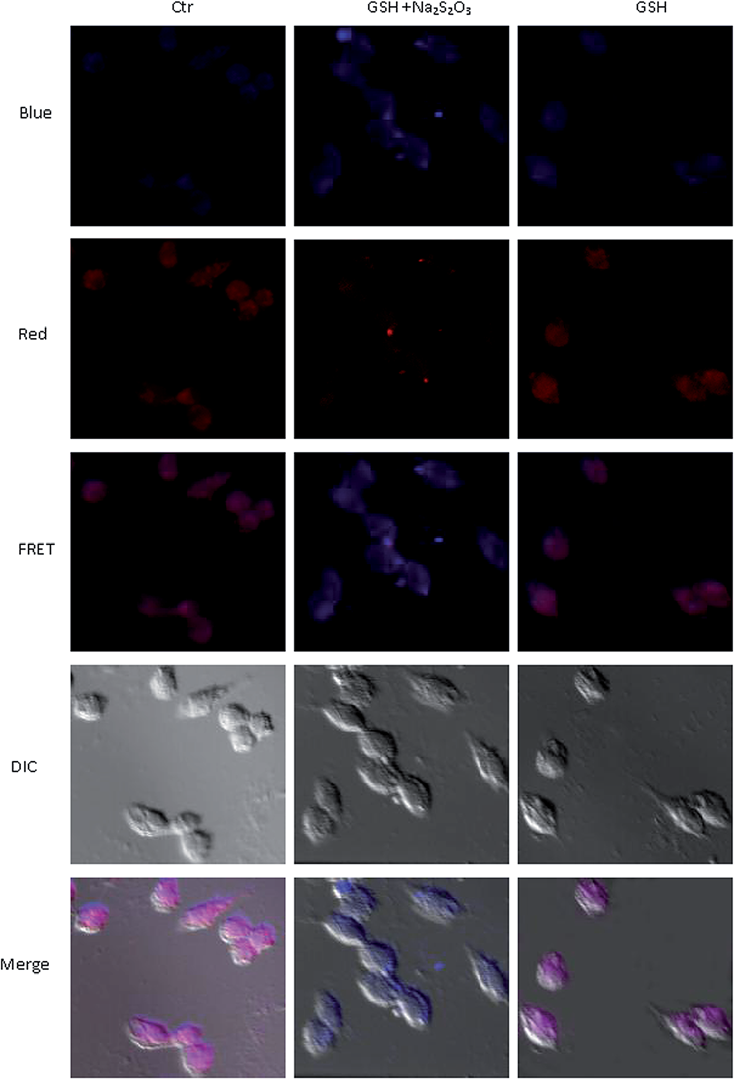
The first line (vertically): glioma cells were incubated with IPIN-SO_2_ (0.1 μM) for 1 h; the second line: glioma cells were incubated with IPIN-SO_2_ (0.1 μM) for 1 h, and then with 0.5 mM GSH and 0.25 mM Na_2_S_2_O_3_ for another 0.5 h; the third line: glioma cells were incubated with probe (0.1 μM) for 1 h, then with 0.5 mM GSH for 0.5 h.

Furthermore, we studied whether the probes could be used to detect exogenous bisulfite in cells. Glioma cells were incubated with probe IPIN-SO_2_ for 1 hour, washed with PBS solution three times, incubated with 0.5 mM GSH (glutathione) and 0.25 mM Na_2_S_2_O_3_ for 0.5 hour, and then photographed with fluorescence inverted microscope to observe obvious fluorescence changes in glioma cells (Fig. 8). On the contrary, no significant fluorescence changes were observed when the glioma cells incubated with IPIN-SO_2_ were incubated only with GSH or Na_2_S_2_O_3_. These results indicate that the probe can detect exogenous bisulfite in glioma cells.

## Conclusions

In summary, a novel FRET-based ratio fluorescence probe of imidazole[1,5-*a*]pyridine substituted hemicyanines has been developed. IPIN-SO_2_ has unique selectivity and high sensitivity (detection limit 0.13 μM) for SO_3_^2−^, which can detect SO_3_^2−^ rapidly (3 min) over a wide pH range of 5 to 10. It is noteworthy that the new ratio fluorescence probe avoids automatic fluorescence, severe self-quenching and fluorescence detection errors. More importantly, the probe has been successfully applied to the identification of exogenous SO_2_ in mitochondria in cells.

## Conflicts of interest

There are no conflicts to declare.

## Supplementary Material

RA-009-C8RA10328C-s001
