# Supplementary material for: A ratiometric fluorescent probe for detection of exogenous mitochondrial SO2 based on a FRET mechanism
Source: RSC Adv. 2019 Mar 18;9(16):8943–8. doi: 10.1039/c8ra10328c (PMC9062019; doi:10.1039/c8ra10328c)
Supplement: RA-009-C8RA10328C-s001 [file RA-009-C8RA10328C-s001.pdf]

Electronic Supplementary Material (ESI) for RSC Advances.
This journal is © The Royal Society of Chemistry 2019

Supplementary data

A ratiometric fluorescent probe for detection of the endogenous mitochondrial SO_2 based on FRET mechanism

Zhiyang Xu, Zhen Chen, Aikun Liu, Ruixue Ji, Xiaoqun Cao, Yanqing Ge*

School of Chemistry and Pharmaceutical Engineering, Taishan Medical University, Taian, Shandong 271016, P. R. China

Corresponding Author: Yanqing Ge
E-mail address: geyanqing2016@126.com
Tel.: +86-538-6229741; Fax: +86-538-6229741

Table of Contents:

Fig. S1 Ultraviolet absorption spectra of **IPIN-SO_2** (10 μM) with the addition of sulfite (0-30 equiv.) in 0.1M PBS (pH = 7.4) buffer solution. **S3**

Fig. S2 Linearity between the fluorescence intensity ratio (F_{475}/F_{580}) and the concentrations of SO_3^{2-} in PBS (pH = 7.4) solution. **S3**

Fig. S3 The photostability of **IPIN-SO_2** in cells. **S4**

Fig. S4 Cytotoxicity assays of **IPIN-SO_2** at different concentrations (1-16 μM) for Glioma cells. **S4**

Fig. S5 Fluorescence images of Glioma cells incubated with **IPIN-SO_2** (0.1 μM) and further incubated with Na_2SO_3 (0, 0.05, 0.5 mM). **S5**

Fig. S6 ^{1}H NMR spectrum of probe **IPIN-SO_2.** **S6**

Fig. S7 ^{13}C NMR spectrum of probe **IPIN-SO_2**. **S6**

Fig. S8 HRMS spectra of **IPIN-SO_2** (A) and **IPIN-SO_2** in the presence of Na_2SO_3 (B). **S7**

Table S1 Comparison of ratiometric fluorescent probes for HSO_3^- **S8**

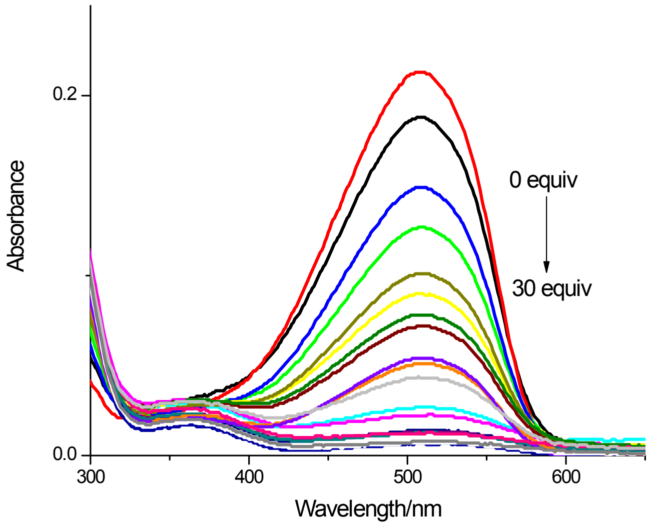

Fig. S1 Ultraviolet absorption spectra of **IPIN-SO$_2$** (10 μM) with the addition of sulfite (0-30 equiv.) in 0.1M PBS (pH = 7.4) buffer solution.

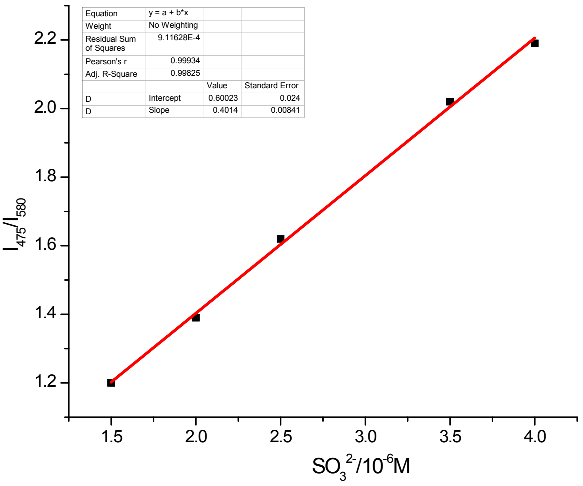

Fig. S2 Linearity between the fluorescence intensity ratio (F_{475}/F_{580}) and the concentrations of SO_3^{2-} in PBS (pH = 7.4) solution.

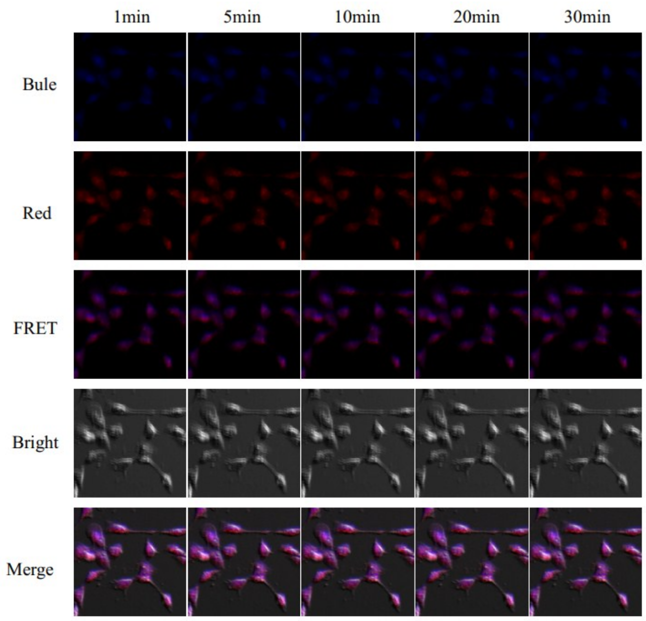

Fig. S3 Photostability for fluorescent images of Glioma cells incubated with 0.1μM **IPIN-SO$_2$** at 37℃. First row: fluorescence images from the blue channel (460/50 nm); second row:fluorescence images at the red channel (605/55 nm); third row: overlay images of the first and second rows; fourth row: bright field images; fifth row: overlay images of the first, second third and fourth rows.

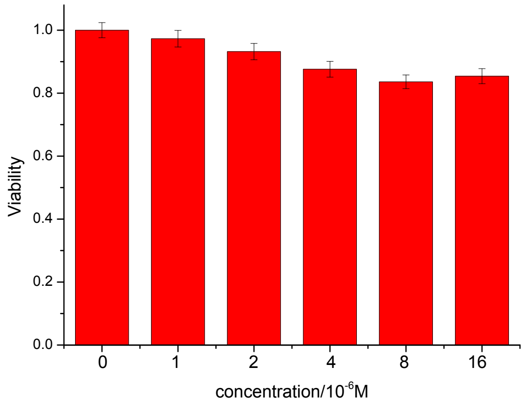

Fig. S4 Cytotoxicity assays of **IPIN-SO$_2$** at different concentrations (1-16 μM) for Glioma cells.

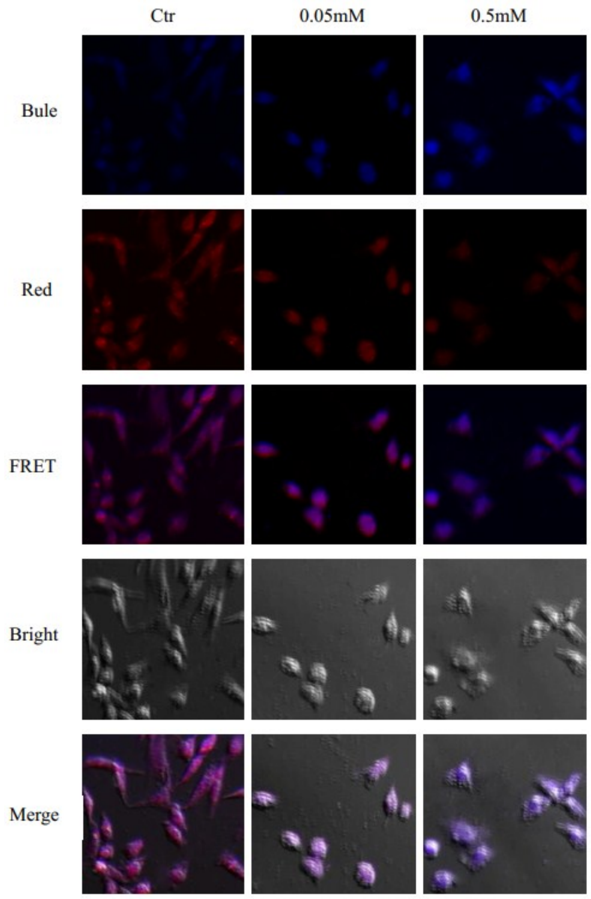

Fig. S5 Fluorescence images of Glioma cells incubated with **IPIN-SO$_2$** (0.1 μM) for 60 min at 37 °C and further incubated with Na_2SO_3 (0, 0.05, 0.5 mM) for 30 min at 37 °C. first row: fluorescence images from the blue channel (460/50 nm); second row: fluorescence images at the red channel (605/55 nm); third row: overlay images of the first and second rows; fourth row: bright field images; fifth row: overlay images of the first, second third and fourth rows.

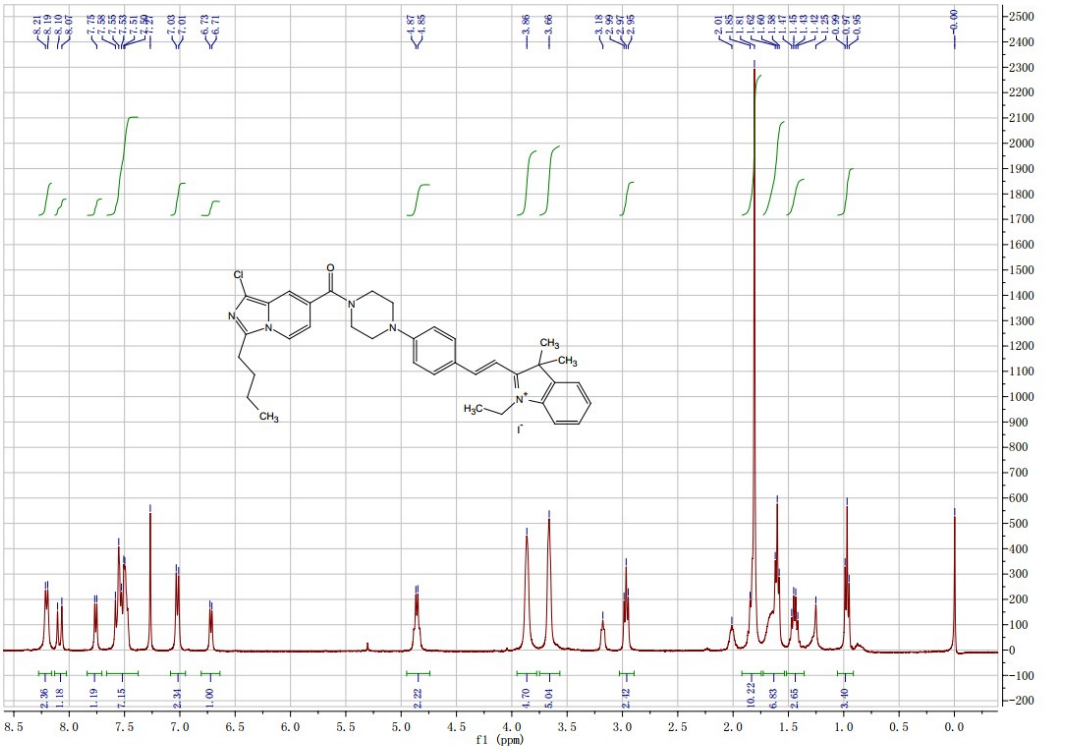

Fig. S6 ^{1}H NMR spectrum of probe **IPIN-SO_2.**

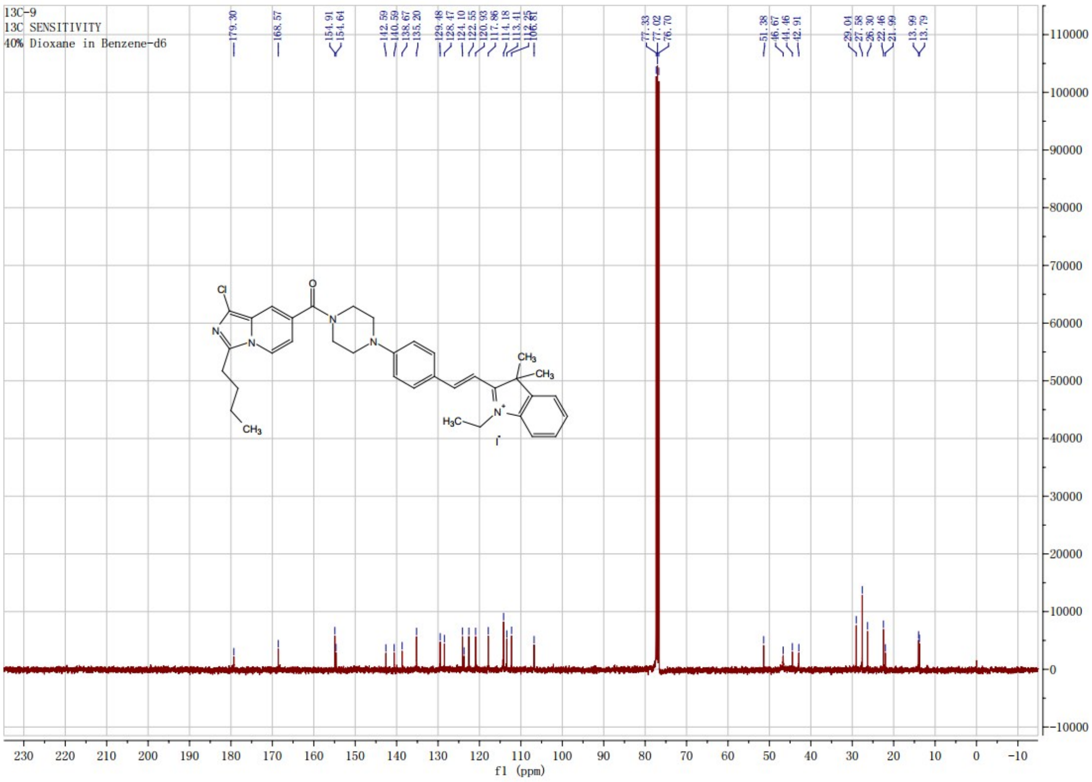

Fig. S7 ^{13}C NMR spectrum of probe **IPIN-SO_2**.

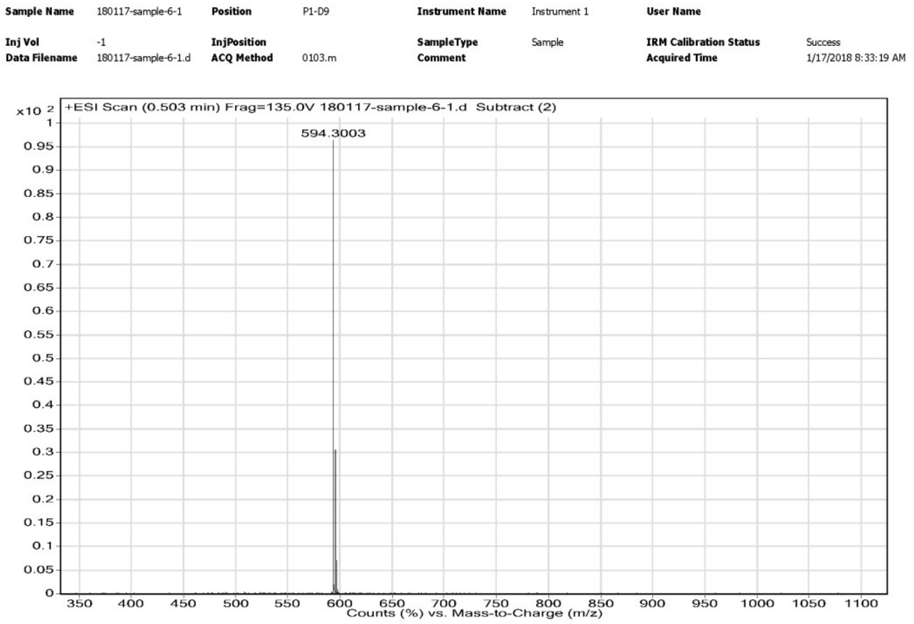

(A)

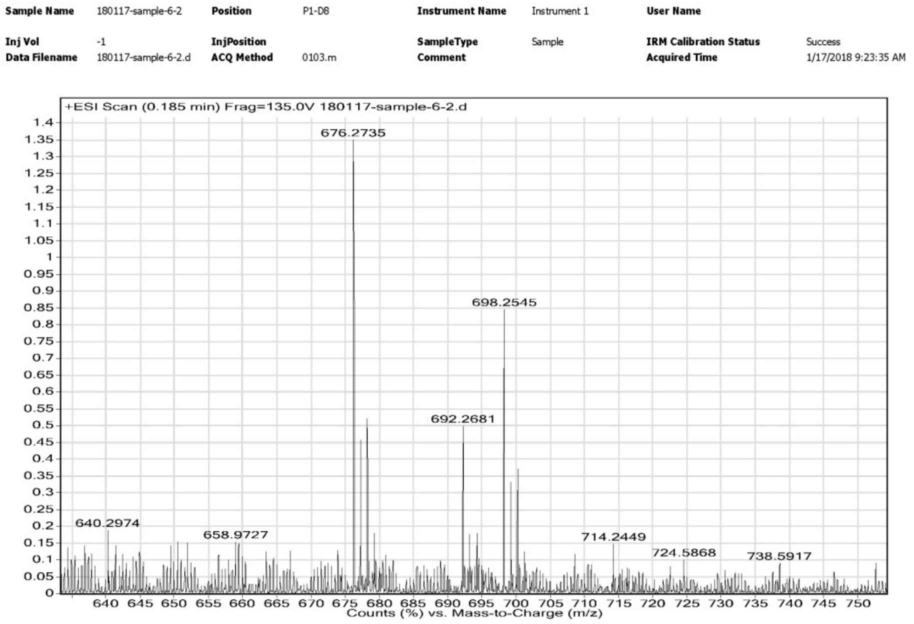

(B)

Fig. S8 HRMS spectra of **IPIN-SO$_2$** (A) and **IPIN-SO$_2$** in the presence of Na_2SO_3 (B).

Table S1 Comparison of ratiometric fluorescent probes for HSO_3^-

Structures	λ_{ex}/nm	λ_{em}/nm	Detection limit	Response time	Ref.
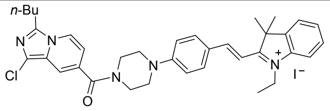	380	475/580	130 nM	180 s	This Work
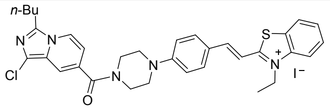	380	477/576	70 nM	1200 s	Dyes Pigments, 2019, 165, 212-216
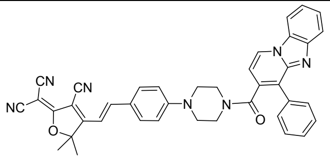	380	465/640	62 nM	120 s	RSC. Adv., 2019, 9, 1147-1150
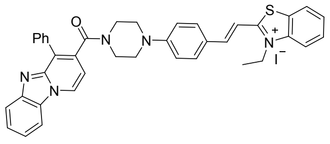	380	470/578	26.7 nM	120 s	Anal. Chim. Acta, 2019, 1053, 148-154
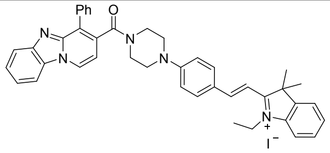	380	470/578	40 nM	120 s	Anal. Chim. Acta, 2019, 1055, 133-139
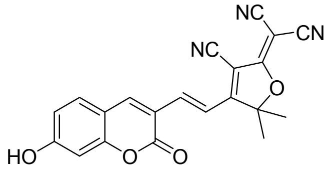	466/580	523/663	0.27 nM	90s	Chem. Commun., 2014, 50, 183
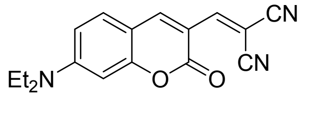	446	480/578	5800 nM	30s	Analyst, 2013, 138, 3018
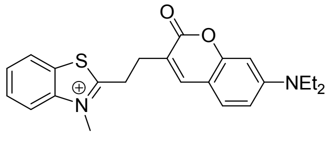	445	478/633	380 nM	300 s	Chem. Commun., 2013, 49, 2637
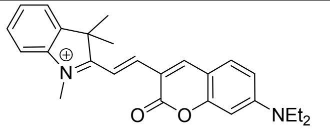	405	480/650	90 nM	180s	Biomaterials, 2015, 56, 1

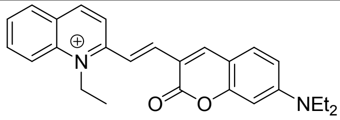	450	518/610	89 nM	900s	Org. Biomol. Chem., 2014, 12, 4637
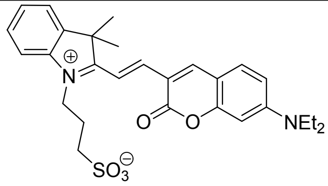	450/550	485/667	27 nM	--	Sens. Actuators, B, 2015, 211, 377
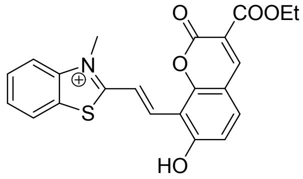	415/500	460/600	100 nM	--	Chem. – Asian J., 2014, 9, 1817
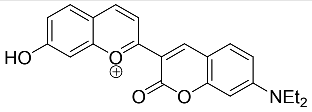	430/605	485/640	34 nM	300s	Sens. Actuators, B, 2015, 206, 268
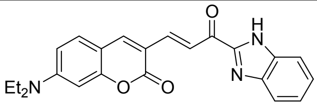	415	485/605	53 nM	3600s	Anal. Chim. Acta, 2015, 888, 138
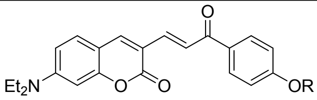	410	465/592	200 nM	$t_{1/2} \approx$ 300s	Anal. Chim. Acta, 2013, 788, 165
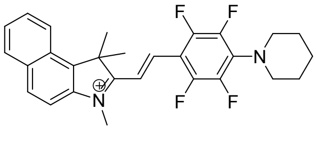	322/470	460/595	3.0 nM	180s	Chem. Commun., 2015, 51, 1154
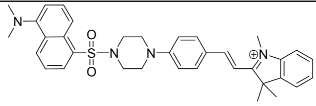	410	530/580	100 nM	120s	Chem. Commun., 2016, 52, 2760
